# Paraoxonase and arylesterase activity of serum PON-1 enzyme in psoriatic patients: a systematic review and meta-analysis

**DOI:** 10.1007/s10238-022-00818-z

**Published:** 2022-03-21

**Authors:** Stefania Bassu, Arduino A. Mangoni, Rosanna Satta, Dario Argiolas, Ciriaco Carru, Angelo Zinellu

**Affiliations:** 1 Department of Biomedical Sciences, University of Sassari, Viale San Pietro 43, 07100 Sassari, Italy; 2 Discipline of Clinical Pharmacology, College of Medicine and Public Health, Flinders University, Adelaide, Australia; 3 Department of Clinical Pharmacology, Flinders Medical Centre, Southern Adelaide Local Health Network, Adelaide, Australia; 4 Department of Clinical, Surgical and Experimental Medicine, University of Sassari, Sassari, Italy

**Keywords:** Arylesterase, Inflammation, Paraoxonase, Psoriasis

## Abstract

Human serum paraoxonase-1 (PON-1) is a critical antioxidant defense system against lipid oxidation. Decreased PON-1 activity has been associated with systemic oxidative stress in several disease states. We conducted a systematic review and meta-analysis of plasma/serum concentrations of PON-1 paraoxonase and arylesterase activity in psoriasis, a chronic immune-mediated and inflammatory skin disease. The electronic databases PubMed, Web of Science, and Scopus were searched from inception to November 2021. In total, 14 studies in 691 psoriatic patients and 724 healthy controls were included in the meta-analysis. Serum paraoxonase activity was significantly lower in psoriatic patients (SMD = − 2.30, 95% CI − 3.17 to − 1.42; *p* < 0.001); however, no significant between-group differences were observed in serum arylesterase activity (SMD = − 0.34, 95% CI − 0.11 to 0.80; *p* = 0.14). The pooled SMD values were not substantially altered in sensitivity analysis. There was no publication bias. In conclusion, our meta-analysis has shown that serum paraoxonase, but not arylesterase, activity is significantly lower in psoriasis, suggesting an impaired antioxidant defense in these patients.

## Introduction

Psoriasis is a chronic and disabling immune-mediated and inflammatory skin disease. The vulgaris form, representing 90% of cases, manifests as erythematous-desquamative plaques that are well delineated from normal skin [1]. The psoriatic plaque is characterized by increased keratinocyte proliferation, dilated dermal vasculature and dermal inflammation with significant leucocyte infiltration. The aetiology of psoriasis is unknown, but genetic factors, immunological mechanisms and metabolic factors have been proposed [1].

Previous studies have demonstrated the presence of alterations in plasma lipids, lipoproteins, lymphocytes, polymorphonuclear leukocytes and platelets in psoriatic patients [2, 3]. In particular, oxidized low-density lipoproteins (ox-LDL) have been observed both in psoriatic skin lesions and serum [4–7]. Accumulation of ox-LDL in psoriatic skin may play an important role in the immune-inflammatory events resulting in progressive skin damage [4].

Furthermore, increased production of reactive oxygen species (ROS) and inflammatory cytokines contribute to the development and progression of psoriatic lesions. Increased concentrations of biochemical markers of lipid peroxidation and a concomitant decrease in cellular and extracellular antioxidants have been demonstrated in subjects with psoriasis [8].

Human serum paraoxonase-1 (PON-1), also known as arylesterase (ARE), is a critical antioxidant defence system against lipid oxidation. It is a glycoprotein of 43–45 kDa and its gene is located in the long arm of chromosome 7 (q21–q22) in humans. PON-1 is an antioxidant and anti-inflammatory calcium-dependent esterase, associated with high-density lipoprotein (HDL), that is used as a marker of lipid peroxidation [9, 10].

PON-1 hydrolyzes the organophosphate substrate paraoxon (paraoxonase activity, EC 3.1.8.1), the toxic metabolite of the insecticide parathion and aromatic esters, such as phenylacetate (arylesterase activity, EC 3.1.1.2) [11].

PON-1 is expressed in the liver and excreted in the blood with the HDL particle [12]. Serum PON-1 hydrolyzes pro-inflammatory oxidized lipids, typically presenting as ox-LDL, and suppresses their atherogenic effects [13].

Decreased PON-1 activity is considered a marker of increased systemic oxidative stress and increased conversion of HDL to a dysfunctional pro-inflammatory and pro-atherogenic state. Not surprisingly, decreased PON-1 activity has been associated with the development of cardiovascular disease [14] and the risk of major adverse cardiovascular events [15].

In order to capture and interpret the available evidence regarding the relationship between psoriasis and PON-1 activity, we conducted a systematic review and meta-analysis of studies reporting plasma/serum concentrations of PON-1 paraoxonase and arylesterase activity in psoriatic patients and control groups.

## Materials and methods

### Search strategy, eligibility criteria and study selection

A systematic search of publications in the electronic databases PubMed, Web of Science and Scopus from inception to November 2021, was conducted using the following terms and their combination: “Paraoxonase” or “PON” or “Paraoxonase-1” or “PON-1” or “arylesterase” and “psoriasis.” Abstracts were screened independently by two investigators to establish relevance. If relevant, the two investigators independently reviewed the full articles. Eligibility criteria were: (i) assessment of paraoxonase and arylesterase activity in plasma or serum; (ii) comparison of subjects with psoriasis and healthy controls (case–control design); (iii) sample size ≥ 10 patients with psoriasis; (iv) English language; and (v) full-text publications. The references of the retrieved articles and reviews were also searched to identify additional studies. Any disagreement between the reviewers was resolved by a third investigator. The risk of bias was assessed using the Joanna Briggs Institute (JBI) Critical Appraisal Checklist for analytical studies. A score of ≥ 5, 4 and < 4 indicated low, moderate and high risk, respectively [16].

The certainty of evidence was assessed using the Grades of Recommendation, Assessment, Development and Evaluation (GRADE) Working Group system. GRADE considers the study design (randomized vs. observational), the risk of bias (JBI checklist), the presence of unexplained heterogeneity, the indirectness of evidence, the imprecision of results (sample size, 95% confidence interval width and threshold crossing), the effect size (small, SMD < 0.5, moderate, SMD 0.5–0.8, and large, SMD > 0.8) [17] and the probability of publication bias [18, 19]. The study complied with the Preferred Reporting Items for Systematic reviews and Meta-Analyses (PRISMA) 2020 statement [20]. The protocol was registered in the International Prospective Register of Systematic Reviews.

### Statistical analysis

Standardized mean differences (SMDs) and 95% confidence intervals (CIs) were used to express forest plots of continuous data and assess differences in PON-1 or ARE concentrations in psoriatic patients vs. healthy controls due to the different units of measurement (U/L, U/mg protein or μmol/ml) used to express the paraoxonase or arylesterase concentrations in studies.

A *p* value < 0.05 was considered statistically significant. When necessary, the means and standard deviations were extrapolated from medians and interquartile values (or ranges), as previously reported by Wan et al. and Hozo et al. [21, 22], or from graphs using the Graph Data Extractor software.

Heterogeneity of SMD across studies was tested by using the *Q* statistic (significance level at *p* < 0.10). The *I*^2^ statistic, a quantitative measure of inconsistency across studies, was also calculated [23, 24]. Statistical heterogeneity was defined as an *I*^2^ statistic value ≥ 50% [24]. A random-effects model was used in presence of high heterogeneity.

Sensitivity analysis was conducted to investigate the influence of each study on the overall risk estimate, by sequentially excluding individual studies [25].

To evaluate the presence of potential publication bias, the associations between study size and magnitude of effect were analyzed by means of Begg’s adjusted rank correlation test and Egger’s regression asymmetry test at the *p* < 0.05 level of significance [26, 27]. The Duval and Tweedie “trim and fill” procedure was carried out to further test and eventually correct the occurrence of publication bias [28]. Univariate meta-regression analyses also were conducted to investigate associations between the effect size and the following parameters: age, gender, publication year, and continent where the study was conducted. Statistical analyses were performed using Stata 14 (STATA Corp., College Station, TX, USA).

## Results

### Systematic research

**Fig. 1:**
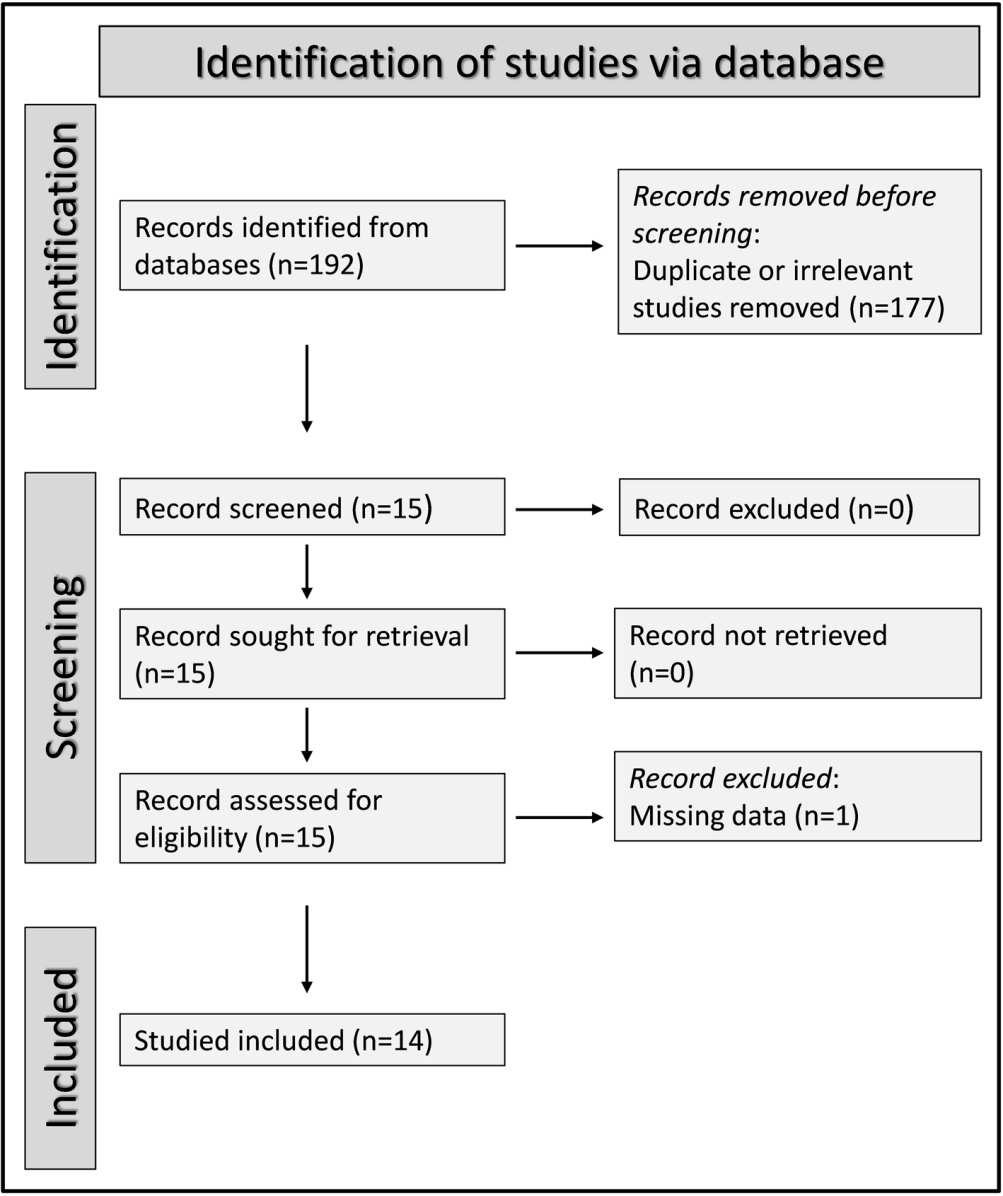
PRISMA 2020 flow diagram

A flowchart describing the screening process is presented in Fig. 1. We initially identified 192 studies. A total of 177 were excluded after the first screening because they were either duplicates or irrelevant. After a full-text revision of remaining 15 articles, one was further excluded because of missing data. Thus, 14 studies in 691 patients with a mean age of 36 years (50% males) and 724 healthy controls with a mean age of 34 years (49% males) were included in the meta-analysis [29–42]. The characteristics of the retrieved studies, published between 2009 and 2021, are presented in Table 1.

**Table 1:** Study characteristics

References, country	Controls					Psoriasis				
	n	Age (years)	Gender (M/F)	PON1 (Mean ± SD)	ARE (Mean ± SD)	n	Age (years)	Gender (M/F)	PON1 (Mean ± SD)	ARE (Mean ± SD)
Toker et al. [29], Turkey	23	30	11/12	27.6 ± 11.8 U/mL	11.6 ± 8.2 U/mL	30	30	12/18	37.1 ± 16.6 U/mL	27.7 ± 16.5 U/mL
Ferretti et al. [30], Italy	25	41	12/13	290 ± 107 U/mL	80 ± 34 U/mL	23	48	10/13	209 ± 103 U/mL	102 ± 32 U/mL
Usta et al. [31], Turkey	25	35	10/15	285 ± 152 U/L	260 ± 38 kU/L	52	37	23/29	194 ± 108 U/L	235 ± 36 kU/L
Asefi et al. [32], Iran	100	36	44/56	–	148 ± 34 U/mL	100	35	43/57	–	126 ± 32 U/mL
Emre et al. [33], Turkey	62	35	29/33	–	215 ± 39 kU/L	54	40	31/23	–	211 ± 22 kU/L
Ramadan et al. [34], Egypt	15	33	4/11	93.7 ± 8.6 U/L	–	15	37	6/9	51.5 ± 8.6 U/L	–
He et al. [35], China	25	43	12/13	14.7 ± 2.4 kU/L	–	25	43	11/14	7.5 ± 1.6 kU/L	–
Holzer et al. [36], Austria	15	43	10/5	352 ± 38 μmol/min/mg	–	15	50	12/3	220 ± 37 μmol/min/mg	–
Houshang et al. [37], Iran	100	36	55/45	153 ± 29 kU/mL	–	100	36	55/45	128 ± 32 kU/mL	–
Husni et al. [38], USA	201	NR	NR	884 ± 773 μmol/min/mL	143 ± 33 μmol/min/mL	84	NR	NR	688 ± 550 μmol/min/mL	111 ± 26 μmol/min/mL
Sorokin et al. [39], USA	20	44	17/3	6.24 ± 3.82 kU/µL	90.8 ± 31.9 U/L	40	50	24/16	8.55 ± 3.21 kU/µL	82.7 ± 23.8 U/L
Bacchetti et al. [40], Italy	48	10	23/25	211 ± 97 U/mL	76.3 ± 18.0 U/mL	52	10	23/29	95 ± 72 U/mL	51.2 ± 21.6 U/mL
Oszukowska et al. [41], Poland	30	NR	14/16	68 ± 6 μmol/mL	–	66	NR	35/31	33 ± 10 μmol/mL	–
Shakoei et al. [42], Iran	35	39	17/18	204 ± 8 U/L	–	35	40	17/18	104 ± 4 U/L	–

ARE, arylesterase; NR, not reported; PON, paraoxonase

### Meta-analysis of paraoxonase activity

#### Study characteristics

Twelve studies in 537 patients (mean age 36 years, 50% males) and 562 healthy controls with a mean age of 34 years (51% males) were evaluated [29–31, 34–42]. Five studies were conducted in Asia [29, 31, 35, 37, 42], four in Europe [30, 36, 40, 41], two in America [38, 39], and one in Africa [34].

#### Risk of bias

The risk of bias was considered low in 10 studies and moderate in the remaining two (Table 2).

**Table 2:** The Joanna Briggs Institute critical appraisal checklist

Study	Were the criteria for inclusion in the sample clearly defined?	Were the study subjects and the setting described in detail?	Was the exposure measured in a valid and reliable way?	Were objective, standard criteria used for measurement of the condition?	Were confounding factors identified?	Were strategies to deal with confounding factors stated?	Were the outcomes measured in a valid and reliable way?	Was appropriate statistical analysis used?	Risk of bias
Toker A	Yes	Yes	Yes	Yes	No	No	Yes	No	Low
Ferretti G	Yes	Yes	Yes	Yes	No	No	Yes	No	Low
Usta M	Yes	Yes	Yes	Yes	Yes	Yes	Yes	Yes	Low
Asefi M	Yes	Yes	Yes	Yes	No	No	Yes	No	Low
Emre S	Yes	Yes	Yes	Yes	No	No	Yes	No	Low
Ramadan S	Yes	Yes	Yes	Yes	No	No	Yes	No	Low
He L	Yes	Yes	Yes	Yes	No	No	Yes	No	Low
Holzer M	No	Yes	Yes	Yes	No	No	Yes	No	Moderate
Houshang N	Yes	Yes	Yes	Yes	No	No	Yes	No	Low
Husni ME	No	Yes	Yes	Yes	No	No	Yes	No	Moderate
Sorokin AV	Yes	Yes	Yes	Yes	No	No	Yes	No	Low
Bacchetti T	Yes	Yes	Yes	Yes	No	No	Yes	No	Low
Oszukowska M	Yes	Yes	Yes	Yes	No	No	Yes	No	Low
Shakoei S	Yes	Yes	Yes	Yes	No	No	Yes	No	Low

#### Results of individual studies and syntheses

**Fig. 2:**
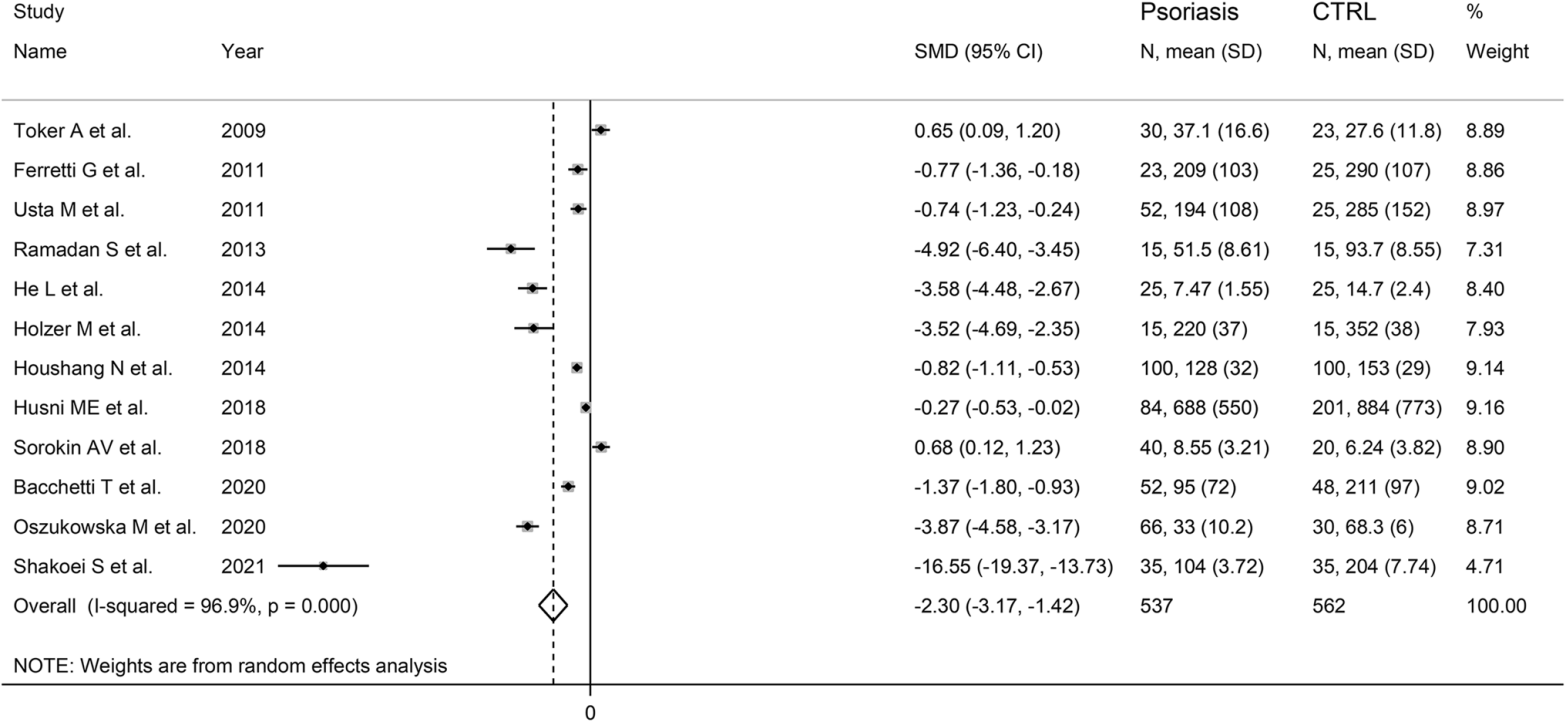
Forest plot of studies examining serum PON values of psoriasis and controls

**Fig. 3:**
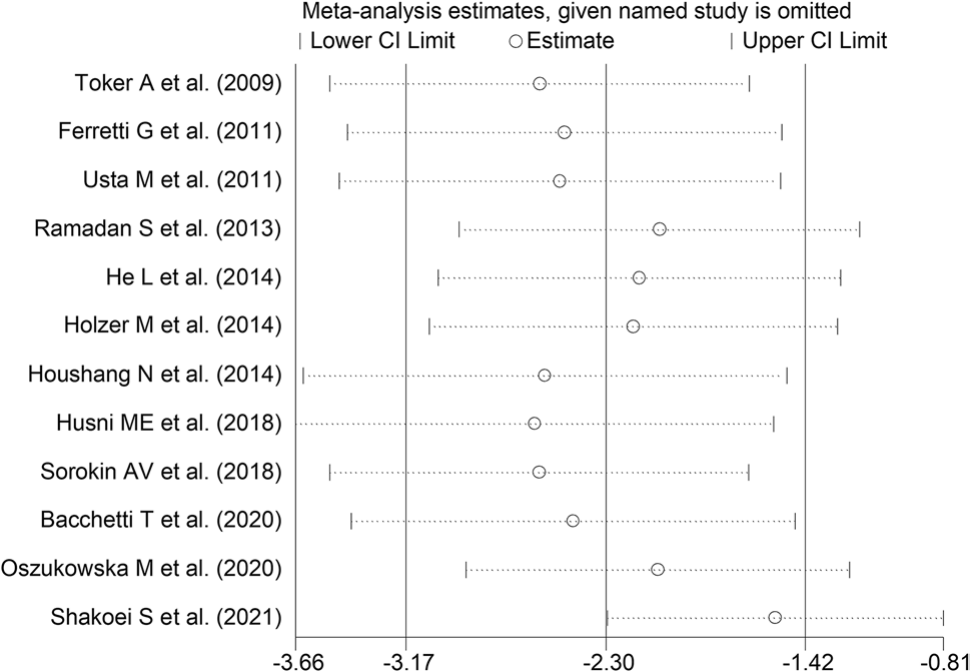
Sensitivity analysis of the association between serum PON values and psoriasis disease. For each study, the displayed effect size (hollow circles) corresponds to an overall effect size computed from a meta-analysis excluding that study

**Fig. 4:**
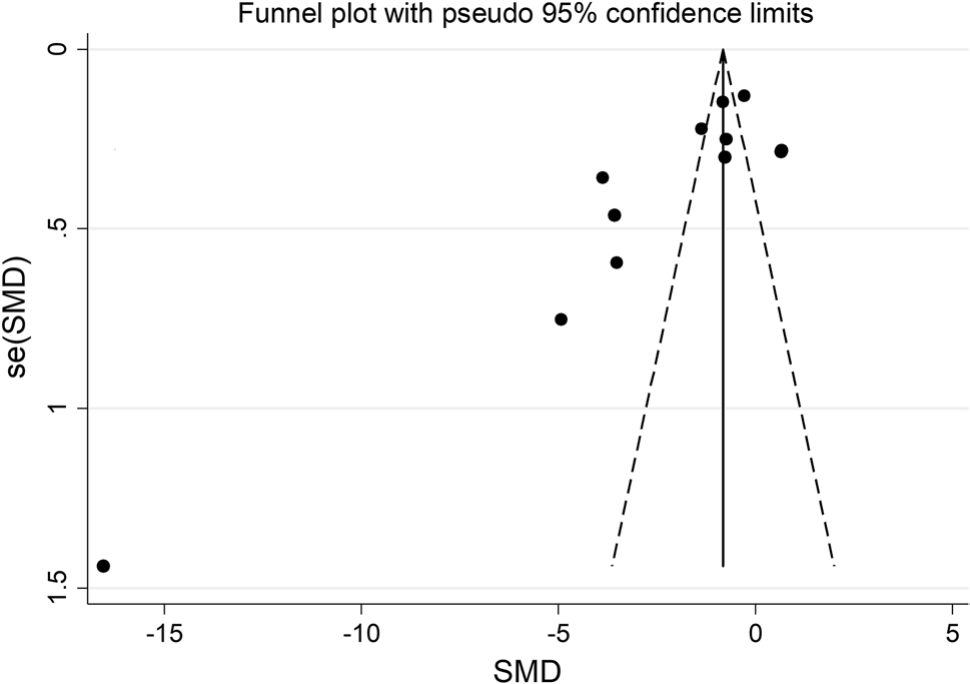
Funnel plot of the 12 retrieved studies evaluating of the association between serum PON concentration and psoriasis disease

The forest plot of PON-1 activity in patients with psoriasis and healthy controls is shown in Fig. 2. In two studies [29, 39], patients had significantly higher PON-1 activity than controls (mean difference range, 0.65 to 0.68). In the remaining ten [30, 31, 34–38, 40–42], patients had lower PON-1 activity than healthy subjects (mean difference range, − 0.27 to -16.65) with a non-significant difference reported only in one study [38]. Extreme heterogeneity was observed (*I*^2^ = 96.9%, *p* < 0.001). Thus, random-effects models were used. Overall, pooled results showed that PON-1 activity was significantly lower in psoriatic patients (SMD = − 2.30, 95% CI − 3.17 to − 1.42; *p* < 0.001). In sensitivity analysis, the corresponding pooled SMD values were not substantially altered when individual studies were sequentially omitted (effect size range, between − 2.61 and − 1.55, Fig. 3). However, funnel plot analysis showed that the study by Shakoei et al. [42] influenced graph symmetry with a possible effect on the effect size (Fig. 4). After removing this study, the SMD, albeit attenuated, remained significant (SMD = − 1.55, 95% CI − 2.29 to − 0.81, *p* < 0.001) with a persistent extreme heterogeneity (*I*^2^ = 95.8%, *p* < 0.001).

#### Publication bias

**Fig. 5:**
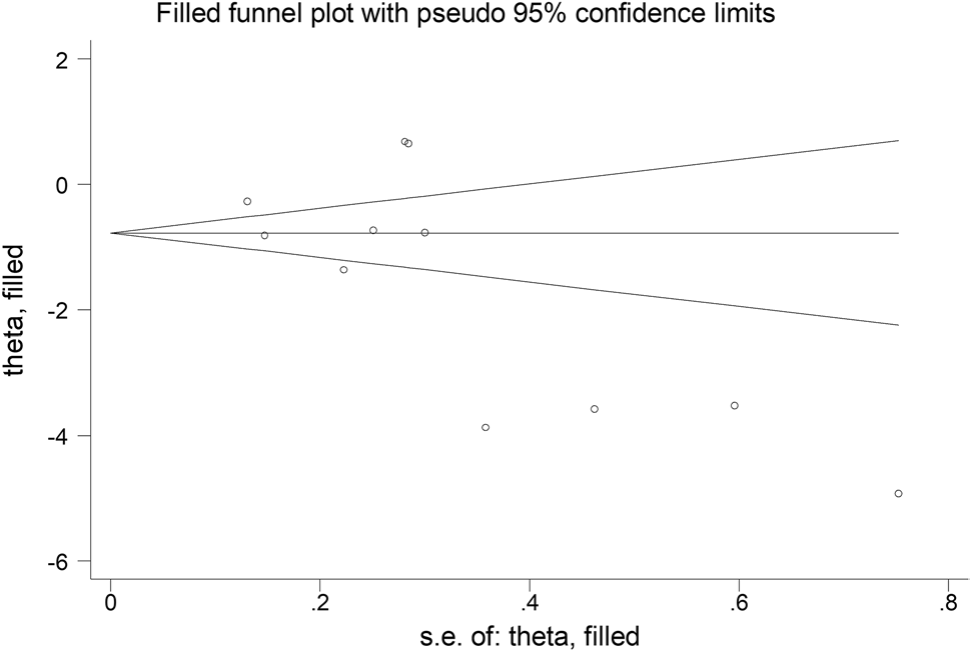
Funnel plot of studies investigating association between serum PON values and psoriasis disease after trimming and filling. Dummy studies and genuine studies are represented by enclosed circles and free circles, respectively

There was no publication bias (Begg’s *p* = 0.12 and Egger tests *p* = 0.06). Accordingly, the “trim-and-fill” method did not identify missing studies to be added in the funnel plot (Fig. 5).

#### Meta-regression and sub-group analysis

**Fig. 6:**
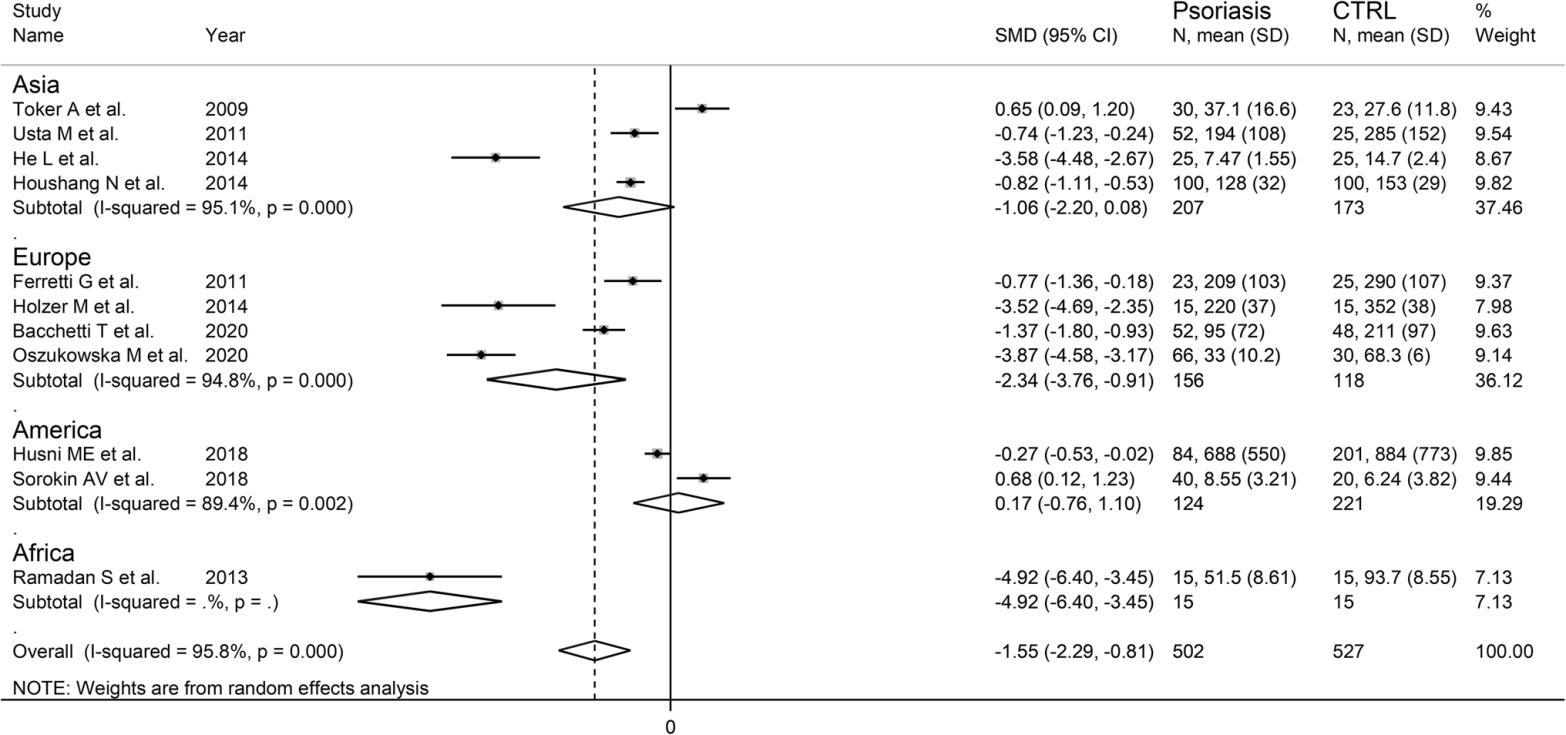
Forest plot of studies examining serum PON concentration in psoriasis and controls according to continent where the study was conducted

In univariate meta-regression, no significant associations were observed between the effect size and age (*t* = − 0.11, *p* = 0.91) and publication year (*t* = − 0.45, *p* = 0.66). However, a significant relation was observed with gender (*t* = − 3.49, *p* = 0.007). In sub-group analysis, reported in Fig. 6, there was a significant psoriasis-associated decrease in serum PON-1 activity in European studies (SMD = − 2.34; 95% CI − 3.76 to − 0.91, *p* = 0.001; *I*^2^ = 94.8%, *p* < 0.001) but not in studies conducted in Asia (SMD = − 1.06; 95% CI − 2.20 to 0.08, *p* = 0.07; *I*^2^ = 95.1%, *p* < 0.001) or America (SMD = 0.17; 95% CI − 0.76 to 1.10, *p* = 0.72; *I*^2^ = 89.4%, *p* = 0.002).

#### Certainty of evidence

The initial level of certainty for PON-1 SMD values was considered low because of the cross-sectional design of the studies (rating 2, ⊕ ⊕⊝⊝). After considering the low risk of bias in 10 out of 12 studies (no rating change required), the extreme and unexplained heterogeneity (downgrade one level), the lack of indirectness (no rating change required), the relatively low imprecision (relatively narrow confidence intervals without threshold crossing, no rating change required), the relatively large effect size (SMD = − 2.30, upgrade one level) and the absence of publication bias (no rating change required), the overall level of certainty remained low (rating 2, ⊕ ⊕⊝⊝).

### Meta-analysis of circulating arylesterase activity

#### Study characteristics

Eight studies in 435 patients (mean age 34 years, 47% males) and 504 healthy controls (mean age 32 years, 48% males) were identified [29–33, 38–40]. Four studies were conducted in Asia [29, 31–33], two in Europe [30, 40] and two in America [38, 39].

#### Risk of bias

The risk of bias was considered low in seven studies and moderate in the remaining one.

#### Results of individual studies and syntheses

**Fig. 7:**
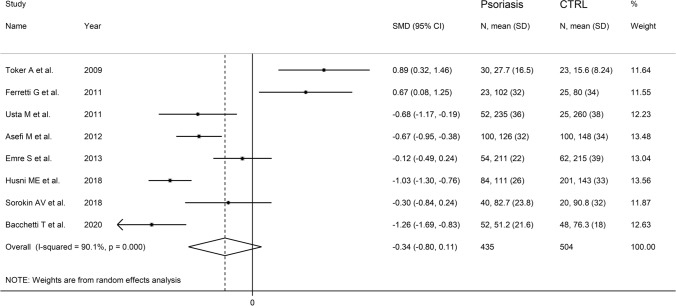
Forest plot of studies examining serum ARE values in psoriasis patients and controls according to continent where the study was conducted

**Fig. 8:**
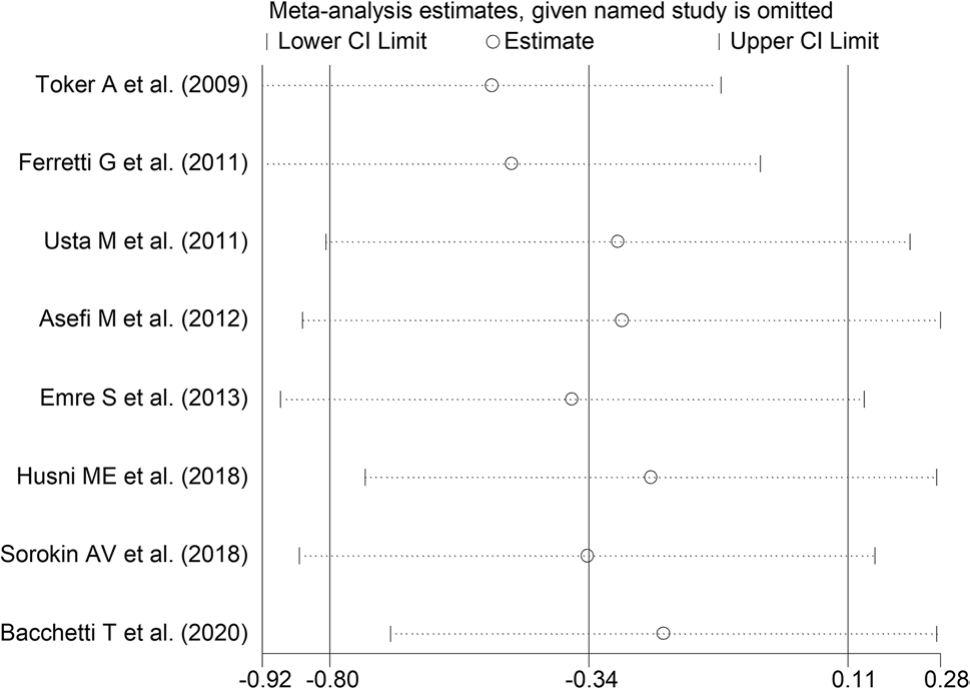
Sensitivity analysis of the association between serum ARE values and psoriasis disease. For each study, the displayed effect size (hollow circles) corresponds to an overall effect size computed from a meta-analysis excluding that study

The forest plot of ARE activity in patients with psoriasis and healthy controls is shown in Fig. 7. In two studies [29, 30], patients had significantly higher ARE activity than controls (mean difference range, 0.67 to 0.89). In the remaining six [31–33, 38–40], patients had lower ARE activity than healthy subjects (mean difference range, − 1.26 to − 0.12) with a significant difference in four studies [31, 32, 38, 40]. Extreme heterogeneity was observed (*I*^2^ = 90.1%, *p* < 0.001). Thus, random-effects models were used. Overall, pooled results showed no significant differences in serum ARE activity between patients and controls (SMD = − 0.34, 95% CI − 0.11 to 0.80; *p* = 0.14). Sensitivity analysis showed that the corresponding pooled SMD values were not altered when individual studies were sequentially omitted (effect size range, between − 0.51 and − 0.28, Fig. 8).

#### Publication bias

Assessment of publication bias was not possible because of the small number of studies.

#### Meta-regression and sub-group analysis

Meta-regression and sub-group analyses were not possible because of the small number of studies.

#### Certainty of evidence

The initial level of certainty for ARE SMD values was considered low because of the cross-sectional design of the studies (rating 2, ⊕ ⊕⊝⊝). After considering the low risk of bias in seven out of eight studies (no rating change required), the extreme and unexplained heterogeneity (downgrade one level), the lack of indirectness (no rating change required), the relatively high imprecision (relatively wide confidence intervals with threshold crossing, downgrade one level, the relatively small effect size (SMD = − 0.34, downgrade one level), and the lack of assessment of publication bias (downgrade one level), the overall level of certainty was downgraded to very low (rating 0, ⊝ ⊝⊝⊝).

## Discussion

In our systematic review and meta-analysis, serum PON-1 paraoxonase, but not arylesterase, activity were significantly lower in psoriatic patients when compared to healthy controls. The relatively large SMD values for PON-1 activity indicate an effect size that is likely to be of biological and/or clinical relevance. Despite the extreme heterogeneity observed, in sensitivity analysis the effect size of PON-1 activity was not significantly affected when each study was in turn removed. The study by Shakoei et al. [42] influenced graph symmetry with a possible effect on the effect size. After removing this study, the SMD remained significant with persistent extreme heterogeneity between studies. Further analyses based on the Begg's and Egger's *t*-tests did not show publication bias. Similarly, the “trim-and-fill” method did not identify potentially missing studies to be added to the funnel plot to ensure symmetry. In meta-regression analysis, only gender was significantly associated with the SMD for PON-1 activity. This observation is in line with studies in type-2 diabetes reporting a gender-specific effect on PON-1 activity [43].

In addition, only European studies detected a significant decrease in serum PON-1 activity in psoriatic patients. As studies have reported that a paraoxonase-1 gene polymorphism significantly affects paraoxanase activity levels in the general population [44], our results suggest that the differences in PON-1 activity might be linked to specific ethnic groups.

Although the exact mechanisms responsible for the lower serum concentrations of PON-1 activity in psoriasis are unclear, the presence of a chronic inflammatory state might play an important role. This proposition is supported by studies reporting that anti-psoriasis treatment was associated with a significant reduction in inflammatory markers and a significant increase in PON-1 activity [45]. However, other studies have shown negative results. Pektas et al*.* [46] did not report any change in PON-l activity in psoriatic patients following 30 phototherapy sessions with UVB narrowband lamps. Similarly, Kilic et al*.* [47] did not observe any increase in PON-l activity in psoriatic patients after 8-week treatment with methotrexate.

Several PON-l gene polymorphisms may affect PON-l activity. One such polymorphism, L55M, involving a change of TTG codon into ATG in exon 3 of the gene, has been associated with reduced PON-l activity [45]. Notably, Asefi et al*.* [48] reported that this polymorphism increases the risk of psoriasis, and its presence is associated with higher circulating concentrations of malondialdehyde, apoB, lp(a) and apoB/apoAI. Further studies are required to investigate the association between specific PON-l gene polymorphisms, PON-l activity and psoriasis.

A reduced PON-1 activity has been observed in several chronic inflammatory disease states. In a large prospective cohort of patients with atherosclerosis undergoing coronary angiography, those with the lowest PON-1 activity had a 3.4 times greater hazard of major cardiovascular events compared to those with the highest PON-1 activity [49]. Impaired PON-1 activity has also been reported to be associated with a higher prevalence of atherosclerotic cardiovascular disease in patients with rheumatoid arthritis and Alzheimer’s disease [50, 51]. Circulating concentrations of leptin, hs-CRP and IL-6 have been found to be significantly associated with PON-1 activity [52]. Furthermore, there is good evidence that PON-1 protects lipids against peroxidation by preventing low-density lipoprotein oxidation, a critical factor involved in the pathogenesis of inflammatory diseases such as atherosclerosis, diabetes and cancer [53].

By our data, no significant differences were observed in serum ARE activity between patients and controls. However, the relatively small number of studies identified prevented meta-regression and sub-group analyses to investigate associations between SMD values and several study and patient characteristics.

Husni et al. [38] showed that both arylesterase and paraoxonase activity are lower in psoriatic patients than controls; however, only arylesterase activity correlated with disease severity in this study. Furthermore, arylesterase activity is less likely to be influenced by genetic factors, suggesting that it may serve as a more sensitive marker of cardiovascular risk than paraoxonase in psoriasis [15]. Further experimental and human studies are required to clarify the role of arylesterase activity in psoriasis.

The extreme between-study heterogeneity represents a limitation of our meta-analysis. However, there was no evidence of publication bias, and the overall effect size was not significantly influenced in sensitivity analyses. In addition, the comprehensive evaluation of the risk of bias and the certainty of evidence according to GRADE significantly strengthens the conclusion of our study.

The lack of significant associations between study, clinical, and demographic characteristics and the SMD, barring the associations between gender and the SMD for PON-1 activity, suggests that other unreported factors might contribute to the observed heterogeneity*.* The use of specific medications influencing PON-1 activity, such as etanercept or methotrexate, was not reported in the studies included in the analysis. Other preanalytical and analytical factors, such as the time required for sample processing or sample storage (different time and temperature), and the protocols and procedures of analytical method might also account for the heterogeneity.

In conclusion, our systematic review and meta-analysis has shown that serum concentrations of PON-1 activity are significantly lower in psoriatic patients. Additional prospective studies are required to investigate the clinical impact PON-1 activity in this group.
